# Antiepileptic drug use among women from the Taiwanese Registry of Epilepsy and Pregnancy: Obstetric complications and fetal malformation outcomes

**DOI:** 10.1371/journal.pone.0189497

**Published:** 2017-12-18

**Authors:** Chang Ching Yeh, Eric C. Lussier, Yi-Ting Sun, Tzuo-Yun Lan, Hsiang-Yu Yu, Tung-Yao Chang

**Affiliations:** 1 Department of Obstetrics & Gynecology, Taipei Veterans General Hospital, Taipei, Taiwan; 2 Institute of Clinical Medicine, National Yang-Ming University, Taipei, Taiwan; 3 Department of Obstetrics and Gynecology, National Yang-Ming University, Taipei, Taiwan; 4 Taiji Clinic, Taipei, Taiwan; 5 Institute of Hospital and Health Care Administration, National Yang-Ming University, Taipei, Taiwan; 6 Department of Public Health, China Medical University, Taichung, Taiwan; 7 Neurology Department, Taipei Veterans General Hospital, Taipei, Taiwan; 8 Neurology Department, National Yang-Ming University, Taipei, Taiwan; University of Modena and Reggio Emilia, ITALY

## Abstract

To investigate antiepileptic drugs (AEDs) prescription and pregnancy outcomes in pregnancies with epilepsy in Taiwan between 2004 and 2015. We retrospectively reviewed data from the Taiwanese Registry of Epilepsy and Pregnancy (TREP). The TREP registry is a voluntary prospective cohort registry, which tracks pregnant women with epilepsy and AED prescription throughout pregnancy, delivery, and early childhood development. All TREP pregnancies (n = 318) that had completed questionnaires up until delivery or had had an unsuccessful pregnancy were analyzed. Over 94.7% of women had been prescribed AEDs during pregnancy, with 69.0% and 25.7% having received monotherapy, or polytherapy, respectively. Among live births, 12 (3.9%) reported malformation. Cesarean section rate was reported higher than usual (54.5%). In 2004, 73.3% of AEDs prescribed were 1^st^ generation, with 1^st^ generation prescription rates falling to only 8.3% of total prescribed in 2015. AED polytherapy also fell during the study period (40.0% to 20.0%). Cesarean sections were found to be higher for women over 35 years, who had generalized epilepsy, or had experienced an obstetric complication during pregnancy term. Binary logistic regression revealed that Cesarean section was associated with maternal complications (OR = 5.11, CI 95% = 1.11–23.51, p = 0.036), while malformations were associated with obstetric complication (OR = 20.46, CI 95% = 4.80–87.21, p<0.001). Both AED risk types were not associated with complications or malformations. Our sample provides a unique insight into the women with epilepsy with AED use during pregnancy. Cesarean section rate was observed to be higher than usual, but malformation rates remained low. Results indicate a decrease in both 1^st^ generation AEDs and proportion of patients receiving polytherapy over the study period. Obstetric complications were associated with Cesarean section. Fetal malformations were significantly associated with obstetric complications. AED risk factors were not significantly associated with either complications or malformations.

## Introduction

Epilepsy is one of the leading neurological causes of burden to the individuals and community, with an annual incidence rate of 40–70 per 100,000 persons per year [1]. Women with Epilepsy (WWE) who are pregnant make up roughly 0.3–0.7% of all pregnancies in developed countries [2]. Generalized epilepsy is associated with more complications in pregnancy and delivery [3]. Further evidence of complications in delivery, WWE had a higher incidence of induction of labor (43.8%) compared to the general populations (17.1%), while the incidence of Cesarean section (CS) was higher in WWE (65.6%), than in the control group (37.1%) [4]. Jandhav et al., also found other maternal health outcomes were similar between WWE and non-epileptic pregnant women (i.e. postpartum hemorrhage, abortion, operative vaginal delivery, preterm delivery, and anemia) [4].

Antiepileptic drugs (AEDs) are a common and effective treatment of seizures in epilepsy, although may pose an increased risk of congenital malformations in pregnancies [5], as well as obstetric complications [6]. Evidence suggests that monotherapy (only taking 1 AED) and newer AEDs (2^nd^ Generation AEDs) are safer in terms of obstetric complications and infant malformations, [7,8]. Although recent findings from population level data in Sweden have found evidence that AEDs are safe.[9] Consensus on whether exposure to AEDs or seizures pose a greater risk has yet to be reached, since studies investigating individual AEDs' effect on fetal development and maternal health are insufficient to make conclusive findings [10]. Recent efforts in developing registries for pregnant WWE has led to a better understanding in the link between AEDs, malformations, and pregnancy complications [5,11,12]. Borthen *et al*.[2] observed epileptic seizures were less harmful, since women with active epilepsy who did not take AEDs had no excessive risk. In contrast, Chen et al. [13] observed seizures were associated with a number of poor pregnancy outcomes in pregnant WWE in the Taiwan setting using the National Health Insurance Research Data (NHIRD). Similarly, Lin *et al*. [14] reported that WWE who had not taken AEDs had a higher risk of having poor birth outcomes. Both studies provided data on pregnancies up until 2003. WWE’s AED use and obstetrical outcomes in Taiwan could be updated to accurately reflect current trends in the region.

The objective of the study was to analyze the Taiwanese Registry of Epilepsy and Pregnancy (TREP) data from 2004–2015. Data from the registry presents a unique look at WWE pregnancies that had detailed seizure, AED use and pregnancy outcomes records. The study updates the latest trends in AED use among WWE in an Asian country with high healthcare accessibility. In addition, we hope to distinguish between the effects of epileptic seizures, AED exposure, and poor obstetric complications.

## Methods

### Design

We retrospectively reviewed data from the prospectively collected *Taiwanese Registry of Epilepsy and Pregnancy* (TREP) data. The TREP was initiated to participate in the *International European Registry of Antiepileptic Drugs and Pregnancy* (EURAP) [15]. The EURAP is a 42 country registry that provides information on WWE pregnancies, AED prescription, and obstetrical outcomes. The TREP registry is a voluntary prospective cohort registry that collects questionnaire responses from WWE upon 5 visits. The questionnaire has been used in a previous preliminary study [16]. Visits were selected to match regular maternal check-up schedules, in order to increase compliance. The 1^st^ visit occurred in early pregnancy, the 2^nd^ visit was at 12 and 13 weeks of gestational age (GA) follow-up, and the 3^rd^ visits recorded responses at 28–32 weeks GA. Following delivery, the 4^th^ questionnaire responses was recorded 7 days after birth, while the last questionnaire was recorded 1 year after birth. During the course of data collection, 50 mothers had more than one pregnancy. We included multiple pregnancies because women’s profile (Age, parity, AED profile, seizure event/frequency, birth outcomes) differed enough to be considered independently.

### Study sample

TREP is a registry collecting data from WWE who: (1) had a pregnancy, (2) were diagnosed with epilepsy, (3) did not have other chronic diseases, (4) were willing to participate in the registry, and (5) were willing to maintain AED intake during the course of the pregnancy as prescribed by a trained physician. Cases with chronic diseases that were excluded included: past history of hypertension, cardiovascular disease or diabetes, women who had history of psychiatric issues. All women that were given a questionnaire also signed an informed consent form. The total TREP pregnancies entered into the registry from 2004 to 2015 was *349*. There were 50 women that entered into the analysis more than once. Although TREP questionnaires recorded all follow-ups until 1 year after birth (5^th^ visit), we chose to include any cases that had at least been followed-up to the 4^th^ questionnaires or had an unsuccessful pregnancy, since we were only interested in maternal health outcomes, and did not focus on child development until 1 year. An additional 30 cases had failed to complete up to the 4^th^ wave, or had incomplete questionnaire information, and were also excluded from the analysis, leaving *319 cases*. Four cases that had malformations were aborted early, 1 case reported Trisomy 21 (S1 Table). Since it is not seen to be clinically linked with epilepsy or AED pharmacologically, we chose to delete the case as including this case may have overestimated malformation outcome. Since many cases were excluded due to missing information, we conducted a sensitivity analysis comparing baseline characteristics between included and excluded samples. No significant differences were observed and thus was not presented here. The final WWE sample was 318 pregnancies. The study was approved by the Research Ethics Committee of Taipei Veterans General Hospital with IRB number 2017-07-017CC.

### Primary measures and covariates

Variables selected from the questionnaire items and responses included: maternal age (continuous and categorical {15–20, 21–25, 26–35, 36–40, ≥41]}, type of epilepsy (Generalized, Localization-related, and Unknown), and maternal history (parity). We also looked at the number of AEDs patients were prescribed (none, monotherapy (1), and polytherapy (≥2)). Previously established categorization of AED by generation were employed [5,17]. 1^st^ generation are AEDs which were developed before 1993, while 2^nd^ generation were developed after 1993. Since some patients had been prescribed both 1^st^ and 2^nd^ generation AEDs, they were coded as having a concurrent prescription. In the analytical stage, in order to isolate exposure to a 1^st^ generation AED we assigned a binary code for those who were exposed to 1^st^ generation and those who were not. Overall seizure frequency was reported for any seizure experienced throughout the pregnancy (Y/N). We also looked more closely at seizures in the first trimester, as previous research indicates that seizures in this trimester are associated with malformations [18]. Three levels of seizures were categorized as: no seizures experienced, <1 seizure/month, ≥1 seizure/month. Gestational age at birth was coded along with normal birth timing: Aborted (≤20 weeks), Preterm Birth (21–36 weeks), Term Birth (37–42 weeks), Post-term Birth (>42 weeks), and late abortion. Having a live birth was coded as a binary category (Y/N). Overall malformations, which included *in utero* malformations for those with early abortions, were identified. Malformations included any congenital physical deformity to external limbs or organ structure, confirmed by physician or ultrasound technician if aborted early or confirmed by pediatrician in post-partum period. For a complete list of malformations detected see S1 Table. Obstetric complications (Y/N) were defined as any WWE who had developed an obstetrical complication in the perinatal period as diagnosed by an obstetrician. For a full list of obstetrical complications see S2 Table. Delivery methods (vaginal delivery, Cesarean (CS), aborted) were also recorded. Cases that had CS were defined into two categories: primary CS (having never had a CS performed previously) or previous CS (having had a CS operation in a previous pregnancy).

### Statistical analysis

The unit of analysis was total pregnancies of WWE. Descriptive data were analyzed using simple counts and percentages (%), and mean and standard deviation for continuous variables. We compared the proportion of number of AEDs prescribed in each year and were plotted with a bar graph. Pearson’s Chi-square (*X*^*2*^) analysis was run between cases that had experienced CS and those who hadn’t. Polytherapy, 1^st^ generation AEDs and CS were coded into binary variables (having exposure/experience or not) for analysis. A binary logistic regression compared factors associated with obstetric complications rate and malformation rate. Factors investigated for obstetric complications included: maternal age (above or below 35 years), overall seizure exposure throughout pregnancy, exposure to polytherapy, exposure to 1^st^ generation AEDs and CS outcome. Factors investigated for fetal malformations included: seizures experienced in 1^st^ Trimester, exposure to polytherapy, exposure to 1^st^ generation AEDs, as well as CS outcome. Both models were adjusted for variables and additionally controlled for epilepsy type and parity. Odds ratios with confidence intervals were reported. A 2-sided p-value of *p <* .*05* was used as a cut-off for significance. All data were analyzed using *SPSS version 21*.*0*.

## Results

Among the 349 pregnancies, 318 (91.1%) had completed a questionnaire in its entirety up until successful delivery, or had had an unsuccessful pregnancy outcomes. *Table 1* shows average maternal age was 29.6 years (SD = ±4.2), with the large majority (73.4%) between 26–35 years at time of pregnancy. The large majority were nulliparous (67.7%). We observed a high prescription rate of AEDs, with 94.7% of patients having been prescribed at least 1 AEDs of which 69.0% and 25.7% had received AED monotherapy, or polytherapy, respectively. The majority received 2^nd^ generation AEDs (44.5%), with 32.9% 1^st^ generation AEDs, and 17.2% receiving concurrent AED prescriptions (1^st^ and 2^nd^ generation AEDs together). Around 47.6% had experienced at least 1 seizure throughout the pregnancy, while within the 1^st^ trimester, 32.6% had experienced some form of seizure. We observed a live birth rate of 96.2%. Overall malformations were found to be 5.0%. Although certain cases had had malformations detected prenatally, some aborted pregnancies (n = 4) left a malformation rate of 3.9% among live births. Obstetric complications, either during pregnancy or in delivery occurred in 4.4% of cases. CS delivery was the most common delivery method (54.5%).

**Table 1:** Descriptives of TREP WWE pregnancies (N = 318^c^).

Variables	*Categories*	*Count/Mean*	*%/SD*
**Age (years)**		**29.6**	**4.2**
Age Group	*15–20*	6	1.9%
	*21–25*	45	14.2%
	*26–35*	233	73.3%
	*36–40*	33	10.4%
	*≥41*	1	.3%
**Epilepsy Type**	*Generalized*	181	56.9%
	*Localization-related*	119	37.4%
	*Unknown or Not Ascertained*	18	5.7%
**Parity**	*Nulliparous*	215	67.6%
	*Primiparous*	85	26.7%
	*Multiparous*	18	5.7%
**No. of AEDs**^**a**^ **prescribed**	*None*	17	5.3%
	*Monotherapy (1)*	219	68.9%
	*Polytherapy (≥ 2)*	82	25.8%
**AED Type**	*1*^*st*^ *Generation*	104	32.7%
	*2*^*nd*^ *Generation*	142	44.7%
	*Cocurrent Prescription*	55	17.3%
	*No AEDs Prescribed*	17	5.3%
**Seizure in Overall Pregnancy**	*No*	167	52.5%
	*Yes*	151	47.5%
**Seizure Events** (1^st^ Trimester)	*No Seizures*	214	67.3%
	*<1 Seizure/month*	56	17.6%
	*>1 Seizure/month*	48	15.1%
**Mean Gestational Age (weeks)**		**38.1**	**4.3**
Gestational Age at birth (weeks)	*Aborted (≤20 weeks)*	10	3.1%
	*Preterm Birth (21–36)*	34	10.7%
	*Term Birth (37–42)*	272	85.5%
	*Post-term Birth (>42)*	2	.6%
**Live Birth**	*No*	11	3.5%
	*Yes*	307	96.5%
**Overall Malformation rate**	*Normal*	303	95.3%
	*Abnormal*	15	4.7%
**Maternal Complications**	*Absent*	304	95.6%
	*Present*	14	4.4%
**Delivery Method**	*Vaginal Delivery*	133	41.8%
	*CS*^*b*^	174	54.7%
	*Aborted or Stillborn*	11	4.4%
**Overall CS** *(N = 174)*	*Previous CS*	46	26.4%
	*Primary CS*	80	46.0%
	*Not Indicated*	48	27.6%

Notes

^a.^AEDs—antiepileptic drug

^b.^CS—ceserean section

c.Trisomy 21 case (n = 1) was excluded, since these malformations are not clinically linked to seizures nor AEDs

**Fig 1:**
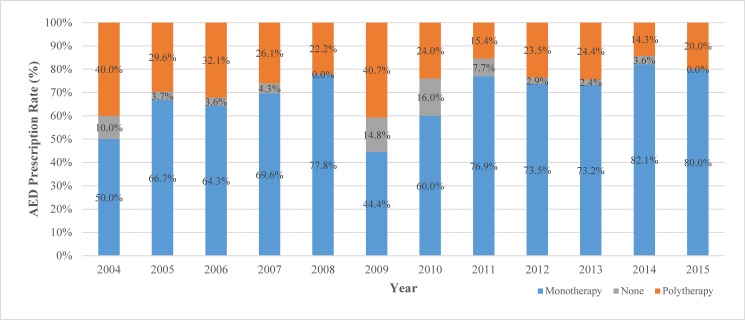
AED monotherapy, polytherapy, and no prescription rates among TREP WWE pregnancies (n = 318).

**Fig 2:**
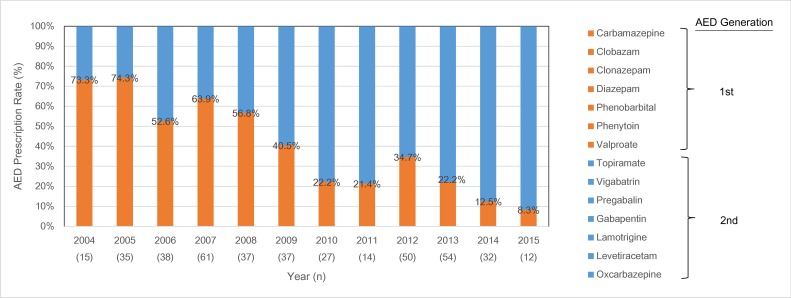
AED prescription by year (2004–2015) and AED generation (1st vs. 2nd) (Total AEDs prescribed: n = 411).

Rates of monotherapy and polytherapy in pregnancy were observed throughout TREP data collection (*Fig 1*). Trends over the course of the study indicate that exposure to polytherapy had steadily decreased, while over the study period from 40% to 20%, while monotherapy had risen from 50% to 80%. Among all AEDs prescribed between 2004 and 2015 (n = 412), variations between 1st generation and 2nd generation AEDs were observed (*Fig 2*). In 2004, 73.3% of AEDs prescribed were 1st generation AEDs, with the 1st generation AED rate steadily falling to 8.3% in 2015.

Next, we categorized pregnancies as either having experienced CS or having experienced a vaginal delivery among those that had a live birth (n = 307) in *Table 2*. Maternal age above 35 years (*X*^*2*^ = 4.347, p = 0.046), seizure experienced throughout pregnancy (*X*^*2*^ = 4.977, p = 0.026), and obstetric complications (*X*^*2*^ = 5.738, p = 0.017), all had significantly higher CS rates compared to the vaginal delivery groups.

**Table 2:** WWE related factors by delivery group (N = 307; aborted: n = 11 excluded).

		Vaginal Delivery (VD)		Cesarean Section (C/S)				
*Variables*	*Categories*	*Count*	*%*	*Count*	*%*	Chi-Square (*X*^*2*^)	*p-value*	*Sig*.
**Maternal Age (years)**						4.347	0.046	*
	<35	120	90.2%	143	82.2%			
	≥35	13	9.8%	31	17.8%			
**Epilepsy Type**						5.697	0.058	
	*Generalized*	69	51.9%	104	59.8%			
	*Localization-related*	51	38.3%	65	37.4%			
	*Unknown or Not Ascertained*	13	9.8%	5	2.9%			
**Seizure Events (1**^**st**^ **Trimester)**						4.915	0.086	
	*No Seizures*	82	61.7%	126	72.4%			
	*<1 Seizure/month*	31	23.3%	24	13.8%			
	*>1 Seizure/month*	20	15.0%	24	13.8%			
**Overall Seizures in Pregnancy**						4.977	0.026	*
	*No*	65	48.9%	101	58.0%			
	*Yes*	68	51.1%	73	42.0%			
**Exposure to AED**^**a**^ **polytherapy**						0.198	0.657	
	*No*	96	72.2%	131	75.3%			
	*Yes*	37	27.8%	43	24.7%			
**Exposure to 1st Generation AED**						1.13	0.288	
	*No*	77	53.1%	82	47.1%			
	*Yes*	68	46.9%	92	52.9%			
**Malformations**						3.614	0.057	
	*Normal*	131	98.5%	164	94.3%			
	*Abnormal*	2	1.5%	10	5.7%			
**Maternal Complications**						5.738	0.017	*
	*Present*	131	98.5%	164	94.3%			
	*Absent*	2	1.5%	10	5.7%			

Notes

^a^AEDs—antiepileptic drug

*p = 0.05

Factors associated with obstetric complications (*Table 3)* and fetal malformations among live births (*Table 4)* were analyzed using a binary logistic regression. Obstetric complications were confirmed to only be associated with CS outcome (OR = 5.11, CI 95% = 1.11–23.51, p = 0.036). Malformations were found to be strongly associated with maternal complications (OR = 20.46, CI 95% = 4.80–87.21, p<0.001). AED polytherapy and 1^st^ generation exposure were not found to be associated with either obstetric complications or fetal malformations.

**Table 3:** Associated factors with maternal complications of WWE^a^ pregnancies, n = 318.

Variables	Category	Complications /N	Complication rate (%)	*aOR* *(CI 95%)*^*d*^^,^^*e*^	P-value	
**Maternal Age (years)**						
	<35	12/273	4.4%	1.00 (Ref.)		
	≥35	2/45	4.4%	1.05 (0.21–5.34)	0.954	
**Overall Seizures in Pregnancy**						
	No	9/167	5.4%	1.00 (Ref.)		
	Yes	5/151	3.3%	0.53 (0.15–1.81)	0.31	
**Exposure to AED**^**b**^ **polytherapy**						
	No	9/236	3.8%	1.00 (Ref.)		
	Yes	5/82	6.1%	2.14 (0.60–7.70)	0.242	
**Exposure to 1st Generation AED**						
	No	6/159	3.8%	1.00 (Ref.)		
	Yes	8/159	5.0%	1.16 (0.36–3.78)	0.805	
**CS**^**c**^ **outcome** (n = 307)						
	No	2/133	1.5%	1.00 (Ref.)		
	Yes	12/174	6.9%	5.01 (1.07–23.41)	0.04	*

Notes.

*p≤0.05

^a.^WWE = women with epilepsy

^b.^AED = antiepileptic drug

^c.^CS = cesarean section

^d.^aOR = adjusted Odds Ratio, CI 95% = confidence interval

^e.^Variables controlled for in model: epilepsy type, parity

**Table 4:** Associated factors with malformation rate among WWE pregnancies, n = 318.

Variables	Category	Malformation /N	Malformation Rate	*aOR*^*c*^^,^^*d*^ *(CI95%)*	P-value	
**Seizures in 1st Trimester**						
	*None*	9/214	4.2%	*1*.*00 (ref*.*)*		
	*<1/month*	2/56	3.6%	0.62 (0.10–3.90)	0.61	
	*≥1/month*	4/48	9.1%	0.49 (0.50–4.89)	0.54	
**Exposure to AED**^**a**^ **polytherapy**						
	No	10/236	4.2%	*1*.*00 (ref*.*)*		
	Yes	5/82	6.1%	0.92 (0.20–4.25)	0.915	
**Exposure to 1st Generation AED**						
	No	5/159	3.1%	*1*.*00 (ref*.*)*		
	Yes	10/159	6.3%	2.08 (0.49–8.78)	0.319	
**Maternal Complications**						
	*No*	10/304	3.3%	*1*.*00 (ref*.*)*		
	*Yes*	5/14	35.7%	20.64 (5.15–99.59)	<0.001	***
**CS**^**b**^ **outcome** (n = 307)						
	No	2/133	1.5%	*1*.*00 (ref*.*)*		
	Yes	10/174	5.7%	2.34 (0.46–12.00)	0.309	

Notes.

***p≤0.001

a. AED = Antiepileptic drug

b. CS = cesarean section

c. aOR = adjusted Odds Ratio, CI 95% = confidence interval

d. Variables controlled for in model: epilepsy type, parity

## Discussion

Our study included WWE pregnancies that had been prescribed AEDs prior to becoming pregnant from 2004–2015 and recorded 318 WWE pregnancies. Improved seizure control is the primary aim of AED prescription in pregnancy, we found relatively similar rates of seizure control during the first trimester compared to the EURAP study *[18]*. Among all pregnancies, 95.7% had taken AEDs throughout their pregnancy term. From a study by Lin et al. [14] they found that NHI data during 2001–2003 had 1182 women coded as epileptic and pregnant. Among them, only 165 (14%) had a record of AEDs prescription and it was estimated that every year there are 83 pregnant women with epilepsy taking AEDs [14]. The annual pregnancies in the TREP ranged between 15 and 47, which accounted for 18%~55% of the WWE pregnancies taking AEDs who were enrolled in this TREP registry. Furthermore, total fertility rate in Taiwan remained at 1.0–1.1 recently and the total population is around 23 million [19]. Our data were characterized by a low total fertility rate and high AEDs exposure during pregnancy which was a reflection of real situation in Taiwan and thus provided an interesting look into a sample with a high exposure to AED use in Taiwan.

Between 2004–2015, 1^st^ generation AEDs were drastically reduced (from 73% to 8%). Starting in 1997 until 2007 a gradual decrease of older AEDs prescriptions and increase in newer generation AEDs had already been observed [5,20,21]. Newer AEDs have been reported to have less side-effects and better tolerability and may be safer in fetal development [22,23]. Polytherapy had also decreased in prevalence during the same timeline (from 40% to 20%). Evidence shows that taking multiple AEDs is associated with a higher risk of malformations and pregnancy complications [7,24]. The reduction of polytherapy over the study period may have been due to improved knowledge of polytherapy risk. In 2009, there was a deviation in AED type prescription trend, with a large spike in polytherapy prescription (40.7%). In Taiwan, Chen et al. [13] and Lin et al. [14] both found AEDs to be safe, while those without AED taken and had seizures saw a higher risk of malformations. We speculate that these papers may have indicated a shift in clinical views and practices, and WWE may have been prescribed higher rates of AEDs. It also possible due to the observational nature of this study, there may have been fluctuations in the data in that year.

Malformation rates were around the average compared with past studies [18,25]. The latest data from EURAP saw a 5.1% malformation rate [18]. *In utero* malformation rates (5.0%) and malformations among live births (3.9%) differed, indicating that cases that had performed advanced screening for severe malformation had been aborted due to early presence of malformations. Among the most recent 588 EURAP cases with major malformations, 110 were detected by ultrasound examination. Out of these 110, there were 51 with induced abortions, four stillbirths, four perinatal deaths and 51 live births. Detection of malformations in our sample were observed in 56% of advanced screenings accurately detecting the presence of malformations in the TREP sample (S1 Table).Seizures during the first trimester have previously been associated with malformations in fetal development, with women with untreated epilepsy having a higher risk of giving birth to children with growth retardation and cognitive dysfunction [26], malformations and live birth rate [23]. Recent improvements in screening methods have allowed obstetricians to detect early malformations and enhance ability to remedy abnormalities or providing mothers with more accurate information to make informed obstetrical decisions [6].

Findings from the TREP sample indicated a higher rate of CS deliveries (54.5%) compared to those in previous studies investigating pregnant WWE: 37.6% [2], 54.3% [5], and 19.8% [10]. CS rates among the general population in Taiwan were also lower than in our study (36.7%) [27]. Most women had never had a previous CS performed. Despite epilepsy not being considered a risk of CS, unless occurring during delivery [3], doctors may have preemptively selected to perform a CS delivery in order to avoid women experiencing seizures in delivery, increasing the occurrence in our sample. Investigating CS we found that maternal age over 35 years, generalized epilepsy and obstetric complications were all more likely to result in a CS delivery. These findings are in accordance with clinical experience and past research, with age [28] and obstetric complications [29], being more likely to lead to CS. Overall experience of at least one epileptic seizure was correlated with higher rates of CS. Past research supports our findings on seizure exposure and risks for pregnancy complications [3,24].

Obstetric complications were found to be significantly associated with CS rate. Complications ranged from early development in pregnancy (ie. Pregnancy induced hypertension) to complications in delivery (ie. Epileptic seizures in delivery) (S2 Table). Obstetric complications have been linked to higher chances of CS [29]. We speculate that since WWE also had an additional complication during pregnancy, physicians may have taken greater precautions, increasing the rate of CS among cases with obstetric complications. None of the TREP cases that had suffered from a complication had resulted in an abortion. In a review by D’Alton *et al*., improvements in fetal care were found to often be accompanied by improvements in maternal healthcare access [30]. WWE pregnancies with complications in early terms would potentially be more likely to attend to healthcare visits which may have prevented further negative effects on the fetal development.

The overall rate of polytherapy use in our cohort was similar to previous studies of AED use in Taiwan (72% vs. 69%) [31]. In contrast to our findings, AED polytherapy and 1^st^ generation have previously been associated with malformations [7,24]. Despite our negative findings for the number of AEDs, our study may have had sample size issues which may have underestimated the effect. Fetal malformations were however highly associated with obstetric complications. Obstetric complications were associated with higher CS rate, however the same association between malformations and CS rate was not detected. Obstetric complication seems to be the most important factor associated with both CS and malformations beyond the effect of age, seizures and AEDs. Epilepsy and AEDs have both previously been found to be risk factors for CS[2] and obstetric complications [8]. Women who had experienced a complication in their pregnancy or in the course of labor, are more likely to be candidates for CS [32], thus potentially explaining the reason for our findings. Association between malformations and obstetric complications was unexpected, since obstetrical complications such as preterm premature rupture of membranes (PPROM) (S2 Table) are not usually associated with malformations. Although recent findings in a large population data study in Sweden found that pregnant WWE had a higher risk of adverse pregnancies or perinatal outcomes, while AED use did not pose a risk, [9] the study pointed out that different AEDs had a range of teratogenic potential. Since we had a small sample size, we aren’t able to conclusively rule out the effect of specific AEDs or seizures on malformations, and it’s possible that WWE complications were influenced by AEDs or seizures and this resulted in higher rate of malformations. Yet it is an interesting finding which deserves further research to investigate the causal pathway between seizures, AEDs, obstetric complications and malformations.

Our study had some limitations. First, registries are important tools in accumulating detailed information especially for diseases with low incidence rates, however they often are not representative of the general population. Our study was cross-sectional with a selected sample of pregnant WWE who were taking AEDs previous to becoming pregnant and were seeking healthcare from a neurologist. Due to selected nature of our sample, our results are not generalizable to the overall population in Taiwan. However, since the occurrence of WWE is a relatively low and those cases who use AEDs during pregnancy are even lower, a selected sample was necessary in order to collect sufficient data. Despite the limitation of bias in our sample, the sample still represents an interesting observation in WWE who had a high AED use throughout pregnancy. Moreover, although including clustered pregnancies as independent cases may pose a risk of biases, many pregnancies from the same mother differed on a number of factors (i.e. age, parity, AED profile, seizure frequency, pregnancy and birth outcomes, etc.) and thus were treated as an independent units of analysis. Second, the TREP registry had accumulated 349 pregnancies in 11 years. The reasons for a small registry number included: low fertility rate, treatment gap of WWE during pregnancy, patient misunderstanding or concealment. [14,33,34]. Third, we only looked at women who had become pregnant. These women would likely differ from WWE who chose not to become pregnant in the severity of epilepsy, would self-select to avoid pregnancy, and may not represent the full spectrum of epilepsy risk. Therefore the results should be generalized to the larger WWE population with caution. Fourth, the questionnaires was self-administered, therefore responses may have been biased in self-reporting for certain characteristics such as seizures experienced during pregnancy and type of epilepsy. Due to patient’s potential gaps in knowledge, some questions may not have been answered accurately.

Despite these limitations, our study provides an interesting look at the Taiwanese sample that had a high rates of AED prescription. Results indicated 1^st^ generation AEDs and polytherapy prescription gradually declining throughout the study period. CS was associated with pregnancies that had suffered an obstetric complication. Despite having a high AED prescription, malformation rates remained low and were not associated with typical AED risk factors. Additionally, CS was associated with obstetric complications, and obstetric complications were associated with malformations. Typical AED risk factors were not associated with higher risks of malformations or complications. Further population level research is needed to explore the links between seizures, AED, obstetric complications and birth outcomes.

## Supporting information

S1 Fig *International European Registry of Antiepileptic Drugs and Pregnancy* (EURAP) questionnaire for pregnant women with epilepsy. In English.
(PDF)

S2 Fig *Taiwanese Registry of Epilepsy and Pregnancy* (TREP) questionnaire for pregnant women with epilepsy. Translated into Mandarin from the EURAP questionnaire (see S1 Fig).
(PDF)

S1 Table Fetal malformation listed by cases (N = 16). *Notes*. ^a^ MCM = major congenital malformation, ^b^ PDA = Patent Ductus Arteriosus, ^c^ DORV = Double outlet right ventricle. ^*d*.^ *Live Birth*: *Y = live birth*, *N = still birth*, *a = aborted*. ^*e*.^ *Ultrasound Screening Performed*: *Y = Yes*, *N = No*, *n/a = not available*. ^*f*.^ *Positive Findings*: *5/9 = 55*.*6% accuracy*. ^g.^ Means reported for live births only. *, **, ***, †, ‡, indicate concurrent maternal complications (S2 Table).
(PDF)

S2 Table Obstetric complications listed by cases (N = 14). *Notes*. ^a.^ CS = Cesarean section, ^b.^ PPROM = Preterm premature rupture of the membranes, ^c.^ n/a = not applicable. ^*d*.^*Live Birth*: *Y = live birth*, *N = still birth*, *a = aborted*. ^e.^ CS rate 11/14 = 78.5%. *, **, ***, †, ‡, indicate concurrent fetal malformations (Table 2).
(PDF)
