# Supplementary material for: Antiepileptic drug use among women from the Taiwanese Registry of Epilepsy and Pregnancy: Obstetric complications and fetal malformation outcomes
Source: PLoS One. 2017 Dec 18;12(12):e0189497. doi: 10.1371/journal.pone.0189497 (PMC5734752; doi:10.1371/journal.pone.0189497)
Supplement: S1 Fig — In English. (PDF) [file pone.0189497.s001.pdf]

SUB-FORM A | SUB-FORM?B | SUB-FORM?C | SUB-FORM?D | SUB-FORM?E

Registration (to be completed as early as possible)

CLICK HERE to enter comments for National Coordinator or Central Registry

Pregnancy identification number 550100355

No.	Field	Value
01	Date of reporting sub-form A (ddmmyyyy)	
02	Identification number ?country	55 Taiwan
03	Identification number ?centre	01 Mackay Memorial Hospital
04	Number of pregnancy of the centre	355
05	When did patient notify her pregnancy? (enter date)	
06	First day of last menstruation (ddmmyyyy)	
07	Reporting physician	
08	Reporting physician email	trep@fetalmedicine.tw
09	Is epilepsy reason for prescribing AED?	
10	If previous question is 0, specify other reason	
11	Is the proband's father affected by epilepsy?	
12	Family name (first 3 letters)	
13	First name (first 3 letters)	
14	Birth date ?mother (ddmmyyyy)	
15	Educational level ?mother	
16	Ethnic background ?mother	
17	Educational level ?father	
18	Ionising radiation exposure 3 months before pregn.	
19	Number of this pregnancy	
20	Number of previous deliveries	○ 88 not ascertained ○ 99 unknown
21	Number of stillborn offspring	○ 88 not ascertained ○ 99 unknown
22	Number of neonatal deaths	○ 88 not ascertained ○ 99 unknown
23	Number of not malformed offspring (enter here also chromosomal abnormalities)	○ 88 not ascertained ○ 99 unknown

24	Number of malformed offspring	○ 88 not ascertained<br>○ 99 unknown
25	Specify malformations or chromosomal abnormalities	
26	Number of spontaneous abortions	○ 88 not ascertained<br>○ 99 unknown
27	Number of induced abortions not due to foetal malformations (enter also chromosomal abnormalities)	○ 88 not ascertained<br>○ 99 unknown
28	Number of induced abortions due to foetal malformations	○ 88 not ascertained<br>○ 99 unknown
29	Specify malformations or chromosomal abnormalities	
30	Type of maternal epilepsy	
31	Aetiology of epilepsy	
32	If an epilepsy syndrome is known, please specify in full, according to the ILAE classification for epilepsy syndromes	
33	Proband family history of congenital malformations	
33 bis	Was patient on AED in previous pregnancies? (Complete only in case of abnormal outcome)	
34	Proband family history of epilepsy	
35	Comments	

Please update the record state Empty

Follow-up at the end of first trimester ( )

CLICK HERE to enter comments for National Coordinator or Central Registry

Pregnancy identification number 550100355

01	Date of reporting sub-form B (ddmmyyyy)	
02	Date of first notification of pregnancy to investigator	
03	Spontaneous abortion	
04	Spontaneous or induced abortion date	
05	Induced termination of pregnancy	
06	Post mortem examination	
07	Specify results (post mortem examination)	
08	First day of last menstruation (ddmmyyyy)	
09	Calculated term date (ddmmyyyy)	
10	Pregnancy duration in completed weeks	
11	Oral contraceptive use during pregnancy	
12	Assisted fertilisation	
13	Cigarette smoking in 1st trimester	
14	Alcohol intake in 1st trimester	
15	Ionising radiation exposure in 1st trimester	
16	Specify other maternal diseases (including relevant infections) in 1st trimester	
17	Folic acid use (dose in 痢) (including folic acid content in multivitamin tablets)	○ 7777 dose unknown<br>○ 8888 not ascertained<br>○ 9999 unknown
18	Start date of folic acid use (ddmmyyyy)	
19	End date of folic acid use (ddmmyyyy)	

20 AED use in 1st trimester (If no, skip next AED fields)

Therapy (click here)

Enter total and largest doses and nr of administrations as they were at the beginning of the 1st trimester (conception)

	AED in 1st trimester (click here for help)		Total daily dose (mg)	Largest single dose (mg)	Nr of administrations	AED start date	AED end date
21	AED1						
22	AED2						
23	AED3						
24	AED4						
25	AED5						

25 bis Was AED total daily dose either increased or decreased from to ?

26 Specify other drugs used during 1st trimester

27 Frequency of generalised tonic-clonic seizures in 1st trimester (including prim/sec generalised)

28 Frequency of other seizures during 1st trimester

29 Status epilepticus during 1st trimester

30 Comments

Please update the record state Empty

Follow-up at the end of second trimester ( )

CLICK HERE to enter comments for National Coordinator or Central Registry

Pregnancy identification number 550100355

01 Date of reporting sub-form C (ddmmyyyy)

02 Date of first notification of pregnancy to investigator

03 Stillbirth

04 Induced termination date or stillbirth date

05 Induced termination of pregnancy

06 Post mortem examination

07 Specify results (post mortem examination)

08 Cigarette smoking in 2nd trimester

09 Alcohol intake in 2nd trimester

10 Specify other maternal diseases (including relevant infections) in 2nd trimester

11 AED use in 2nd trimester (if no, skip next AED fields)

Therapy (click here)

Enter total dose and nr of administrations as they were at the beginning of the 2nd trimester

	AED in 2nd trimester. Fill in even if unchanged. Click here for help.	Total daily dose (mg)	Nr of administrations	AED start date	AED end date
12	AED1				
13	AED2				
14	AED3				
15	AED4				
16	AED5				

16 bis Was AED total daily dose either increased or decreased from to ?

17 Specify other drugs used during 2nd trimester

18 Frequency of generalised tonic-clonic seizures during 2nd trimester (including prim/sec generalised)

19	Frequency of other seizures during 2nd trimester
20	Status epilepticus during 2nd trimester
21	Malformation directed ultrasound
22	Date of ultrasound (ddmmyyyy)
23	Specify result of ultrasound
24	Chorionic villus sampling
25	Amniocentesis
26	Results of amniocentesis AFP in 痢/ml
27	Results of chorionic villus sampling r amniotic cells including karyotype
28	Results of maternal serum AFP in 痢/ml
29	Obstetric complications
30	Comments

Please update the record state Empty

Follow-up at birth ( )

To be completed within three months after birth

CLICK HERE to enter comments for National Coordinator or Central Registry

Pregnancy identification number 550100355

01 Date of reporting sub-form D (ddmmyyyy)

02 Date of first notification of pregnancy to investigator

03 Cigarette smoking in 3rd trimester

04 Alcohol intake in 3rd trimester

05 Specify other maternal diseases (including relevant infections) in 3rd trimester

06 AED use in 3rd trimester (if no, skip next AED fields)

Therapy (click here)

Enter total dose and nr of administrations as they were at the beginning of the 3rd trimester

	AED in 3rd trimester. Fill in even if unchanged. Click here for help.	Total daily dose (mg)	Nr of administrations	AED start date	AED end date
07	AED1				
08	AED2				
09	AED3				
10	AED4				
11	AED5				

11 bis Was AED total daily dose either increased or decreased from to ?

12 Specify other drugs used during 3rd trimester

13 Frequency of generalised tonic-clonic seizures during 3rd trimester (including prim/sec generalised)

14 Frequency of other seizures during 3rd trimester

15 Status epilepticus during 3rd trimester

16 Obstetric complications

17 Birth date ?proband (child out of this pregnancy) (ddmmyyyy) Gestational age in weeks

18 Sex

19	Place of birth ?proband	
20	Delivery of proband	
21	Single or multiple birth (for multiple births, fill in one form for each child)	
22	Sezures during delivery (including status epilepticus)	
23	Apgar score proband at 1 minute	○ 88 not ascertained<br>○ 99 unknown
24	Apgar score proband at 5 minute	○ 88 not ascertained<br>○ 99 unknown
25	Weight of proband at birth in grams	○ 8888 not ascertained<br>○ 9999 unknown
26	Length of proband at birth in cm	○ 88 not ascertained<br>○ 99 unknown
27	Occipital-frontal head circumference of proband at birth in cm	○ 88 not ascertained<br>○ 99 unknown
28	Perinatal death of proband	
29	If yes, specify date (ddmmyyyy)	
30	If yes, specify cause of perinatal death of proband	
31	Congenital malformations	
32	If congenital malformations yes, specify in detail (including date at detection)	
33	Comments	

Please update the record state Empty

Follow-up at the age of one year

CLICK HERE to enter comments for National Coordinator or Central Registry

Pregnancy identification number: 550100355

01	Date of reporting sub-form E (ddmmyyyy)	
02	Date of first notification of pregnancy to investigator	
03	Method of ascertainment	
04	Death during first year of life	
05	If yes, specify cause	
06	Congenital malformations detected postnatally	
07	If yes, specify characteristics in details	
08	Specify age in months at detection of malformation	
09	Hospital admission during 1st year of life	
10	If yes, specify the number of hospitalization and the reasons	
11	Comments	

Please update the record state: Empty
