# Supplementary material for: Antiepileptic drug use among women from the Taiwanese Registry of Epilepsy and Pregnancy: Obstetric complications and fetal malformation outcomes
Source: PLoS One. 2017 Dec 18;12(12):e0189497. doi: 10.1371/journal.pone.0189497 (PMC5734752; doi:10.1371/journal.pone.0189497)
Supplement: S2 Fig — Translated into Mandarin from the EURAP questionnaire (see S1 Fig). (PDF) [file pone.0189497.s002.pdf]

台灣癲癇妊娠登錄系統收案同意書

親愛的媽媽　您好：

為提供您更好的醫療品質及改進空間及了解抗癲癇藥物對胎兒之影響，我們關心您及寶寶可能遭遇的問題，為了解您及寶寶的需求，台灣癲癇妊娠登錄系統問卷追蹤期間分別為：A 表（第一次產檢時）、B 表（12～13 週產檢時）、C 表（抽 B 型肝炎時）、D 表（生產完後 7 天內）、E 表（寶寶週歲時）五個階段、期間醫護人員將協助您填寫問卷，如果您同意參與本登錄系統，請您配合醫護人員之電話追蹤，並按時協助填寫此五份問卷內容。在追蹤過程中您所提供的的資料僅用於研究分析，而您的名字及所有相關資料我們將絕對保密，且絕不做其他用途，保證不會對外公開您的資料。

在您懷孕與育兒的過程中，若有任何問題，歡迎您隨時來電洽詢或以電子郵件方式詢問（trep@fetalmedicine.tw），我們將盡力解答您的問題。

在此致上最誠摯的謝意，敬祝您

身體健康　萬事如意

台灣癲癇妊娠登錄系統　敬上

簽名：____________

日期：________________________

台灣癲癇妊娠登錄系統（A 表）

（第一次產檢時填寫）

※ 填表前請先確定您有填寫收案同意書

※ 本問卷只做個案管理及追蹤，並保證不會將資料外流，請放心填寫

為進一步了解您的健康情形，以便在懷孕的過程中提供更佳的臨床處置，請務必確實回答以下問題：

一、基本資料

病歷號碼：________________　　姓名：______________

身分證字號：_______________　　□本國籍　□外國籍：___________________

電話：(O)_________________　　（請至少留一個電話）

(H)_________________

手機：_______________________

通訊地址：______________________________________

電子郵件帳號：___________________________________

二、家族史及過去疾病史

01.A 表登錄日期：西元______年____月_____日

02.神經內科就診醫院：________________　　神經內科主治醫師：__________________

03.婦產科就診醫院：_________________　　婦產科主治醫師：___________________

04.專責小兒科就診醫院：________________　　專責小兒科就診醫師：________________

05.個案登錄日期：西元______年____月____日

06.最後一次月經日期：西元______年_____月_____日

07.預產期：西元______年____月_____日

08.目前懷孕週數：_______

09.是否因罹患癲癇而服用抗癲癇藥物：　□否　　□是（請跳答 11 題）

10.其它服用抗癲癇藥物的原因：____

11.男方是否亦有癲癇病史：□否　□是　□不確定　□不知道

12.姓氏英文拼音：_______

13.名字英文拼音：_____________

14.孕婦出生日期：西元______年____月_____日

15.孕婦教育程度：□大學及以上　□中學　□小學　□無　□不確定　□不知道

16.孕婦族裔：□白種人 □北非人 □黑人 □亞洲人 □原住民 □大洋洲
□混血兒 □其他 □不確定 □不知道

17.男方教育程度：□大學及以上 □中學 □小學 □無 □不確定 □不知道

18.懷孕之前的三個月是否曾照射 X 光：□否 □是 □不確定 □不知道

19.曾經懷孕次數（包含此次）：□第一次 □第二次 □第三次 □第四次
□第五次 □大於五次 □不確定 □不知道

20.曾經生產次數（>22 週）：_____ （請同時將生產結果填答於 21~25 題）
□不確定 □不知道

21.曾死胎數：______ □不確定 □不知道

22.曾新生兒死亡數(出生後≦七天內死亡)：______ □不確定 □不知道

23.曾生產正常嬰兒數：_______ □不確定 □不知道

24.曾生產的畸形兒數：_______ □不確定 □不知道

25.描述畸胎類型（若 24 題答 0，此題免填）：___________________________________

26.曾自然流產數（妊娠未滿 23 週）：_____次，懷孕____週時 □不確定 □不知道

27.曾因孕婦適應症人工流產數：_______次，懷孕____週時 □不確定 □不知道

28.曾因胎兒畸形引起之人工流產數：____次，懷孕____週時 □不確定 □不知道

29.描述畸胎類型（若 28 題答 0，此題免填）：____

30.孕婦癲癇類型：□一般性 □局部性 □尚未診斷 □不確定 □不知道

31.癲癇致病機轉：□原發性的 □有症狀的 □不明原因的 □不確定 □不知道

32.ILAE 分類：____

33.家族畸胎史：□無（請跳答 34 題）
□媽媽 □爸爸 □姐妹 □兄弟 □兄弟姐妹 □雙胞胎 □不確定

33 bis（若前胎為畸胎才需要填寫本題）
和本次懷孕比較，前胎懷孕期間是否有服用抗癲癇藥物：
□是(使用相同 AED) □是(使用不同的 AED) □否 □不確定 □不知道

34.家族癲癇史：□無 □媽媽 □爸爸 □姐妹 □兄弟 □兄弟姐妹 □不確定

35.備註：（請簡述癲癇發病史及相關檢查結果，包括幾歲時第一次發作，發作的原因，發作時的情況，曾做過的檢查及檢查結果）

____
____
____
____
____

台灣癲癇妊娠登錄系統（B 表）

（12~13 週產檢時填寫）

※ 填表前請先確定您有填寫過收案同意書

※ 本問卷只做個案管理及追蹤，並保證不會將資料外流，請放心填寫

身份證字號：______________________

為進一步了解您的健康情形，以便在懷孕的過程中提供更佳的臨床處置，請務必確實回答以下問題：

01. B 表登錄日期：西元_______年____月____日

02.個案登錄日期：（免填）

03.自然流產：□否（請跳答 05 題） □是 □不確定 □不知道

04.流產日期：西元_________年_____月_____日

05. 人工終止妊娠：□否 （勾選否或胎兒異常以外原因請跳答 11 題）
原因：□胎兒異常 □母親適應症 □社會因素 □其他 □不確定 □不知道

06.胎兒異常類型：□未檢查 □染色體異常 □畸形
□染色體異常且畸形 □不確定 □不知道

07.描述引產後檢查結果：
____

08.最後一次月經日期：（免填）

09.預產期：（免填）

10.目前懷孕週數：（免填）

11.妊娠中服用避孕藥物：□否 □是 □不確定 □不知道

12.人工受孕：□否 □是（自然受孕） □不確定 □不知道

13.第一孕期內抽煙：□無 □1-10 隻/天 □11-20 隻/天 □ >20 隻/天 □不確定 □不知道

14.第一孕期內飲酒：□無 □<1 杯/天 □<3 杯/天 □3-6 杯/天 □>6 杯/天 □不確定 □不知道

15.第一孕期內照射 X 光：□否 □是 □不確定 □不知道

16.詳述其他第一孕期內孕婦曾有疾病：
____

17.葉酸的補充：____mg □有服用葉酸但不知道劑量 □不確定 □不知道

18.開始補充葉酸的日期：西元_____年____月____日

19.停止補充葉酸的日期：西元_________年_____月_____日

20.第一孕期內是否服用抗癲癇藥物：□否（請跳答 26 題） □是（請於 21~25 題說明）
□不確定（請跳答 26 題） □不知道（請跳答 26 題）

抗癲癇藥物（AED）	學名	每日服用總劑量(mg)	單次服用最高劑量(mg)	每日服用次數	開始服用日期 (yyyy/mm/dd)	結束服用日期 (yyyy/mm/dd)
21.AED1						
22.AED2						
23.AED3						
24.AED4						
25.AED5						
25 bis 第一孕期內是否有更換抗癲癇藥物種類或劑量？□無 □是 □不確定 □不知道						
*****若曾變更、增加或減少抗癲癇藥物的種類或劑量，皆須記錄該種類或劑量的開始及結束服用日期*****						

26.第一孕期使用的其它藥物（若無則免填）：______________________________

27.第一孕期時全身性大發作次數：（發作時為全身抽蓄、牙關緊閉等情形）
□無 □<1 次/月 □每月都有 □每週都有 □>每週 □每天都有
□其他 □不確定 □不知道

28.第一孕期時一般性發作次數：（發作時為失神性或局部抽蓄等情形）
□無 □<1 次/月 □每月都有 □每週都有 □>每週 □每天都有
□其他 □不確定 □不知道

29. 第一孕期時是否曾發生癲癇重積的情形：□無 □無抽搐 □有抽搐 □不確定 □不知道
（全身性大發作時間超過 5 分鐘，且反覆發作，反覆發作的過程中皆無恢復意識）

30.備註（前 1~29 題若需補充說明，請簡述於此）：

____

____

____

____

台灣癲癇妊娠登錄系統（C 表）

（抽 B 型肝炎時填寫）

※ 填表前請先確定您有填寫過收案同意書

※ 本問卷只做個案管理及追蹤，並保證不會將資料外流，請放心填寫

身份證字號：Q223038044

為進一步了解您的健康情形，以便在懷孕的過程中提供更佳的臨床處置，請務必確實回答以下問題：

01. C 表登錄日期：西元_____年___月___日

02.個案登錄日期：（免填）

03.死產：□否（請跳答 5 題）　□是　□不確定　□不知道

04.終止妊娠或死產日期：西元_________年_____月_____日

05.人工終止妊娠：□否　（勾選否或胎兒異常以外原因請跳答 08 題）
　原因：□胎兒異常　□母親適應症　□社會因素　□其他　□不確定　□不知道

06.胎兒異常類型：□未檢查　□染色體異常　□畸形
　□染色體異常且畸形　□不確定　□不知道

07.描述引產後檢查結果：（若未檢查則免填）________________________

08.第二孕期內抽煙：□無　□1-10 隻/天　□11-20 隻/天　□ >20 隻/天　□不確定　□不知道

09.第二孕期內飲酒：□無　□<1 杯/天　□<3 杯/天　□3-6 杯/天　□>6 杯/天　□不確定□不知道

10.詳述其他第二孕期內疾病及感染：（若無則免填）________________________

11.第二孕期內是否服用抗癲癇藥物：□否　□是　□不確定　□不知道

抗癲癇藥物（AED）	學名	每日服用總劑量(mg)	每日服用次數	開始服用日期 (yyyy/mm/dd)	結束服用日期 (yyyy/mm/dd)
12.AED1					
13.AED2					
14.AED3					
15.AED4					
16.AED5					

16 bis 第一孕期內是否有更換抗癲癇藥物種類或劑量？□無　□是　□不確定　□不知道

*****若曾變更、增加或減少抗癲癇藥物的種類或劑量，皆須記錄該種類或劑量的開始及結束服用日期*****

17.第二孕期使用的其它藥物：（若無則免填）________________________

18.第一孕期時全身性大發作次數：（發作時為全身抽蓄、牙關緊閉等情形）
□無 □<1 次/月 □每月都有 □每週都有 □>每週 □每天都有
□其他 □不確定 □不知道

19.第一孕期時一般性發作次數：（發作時為失神性或局部抽蓄等情形）
□無 □<1 次/月 □每月都有 □每週都有 □>每週 □每天都有
□其他 □不確定 □不知道

20. 第一孕期時是否曾發生癲癇重積的情形：□無 □無抽搐 □有抽搐 □不確定 □不知道
（全身性大發作時間超過 5 分鐘，且反覆發作，反覆發作的過程中皆無恢復意識）

21.接受高層次超音波檢查：□沒有檢查 □是，且正常 □是，但畸形
□是，畸形外的異常 □不確定 □不知道

22.高層次超音波檢查日期：西元____年____月_____日

胎兒數目：____（胎兒編號：□A □B □C □D □E，並以編號填寫下列項目）

超音波檢查胎位：___頭位 ___橫位 ___臀位 ___其他：________________

超音波檢查位置：___上 ___下 ___左 ___右 ___右上 ___右下 ___左上 ___左下
___中間 ___其他：________________

超音波檢查性別：____男 ___女 ___不確定 ___不知道

23.描述高層次超音波檢查結果：（如有異常才需填寫）

____

24.是否接受絨毛膜採樣：□否 □是，且胎兒染色體正常 □是，但胎兒染色體異常
□不確定 □不知道

25.是否接受羊膜穿刺採樣：□否 □是，且胎兒染色體正常 □是，但胎兒染色體異常
□不確定 □不知道

26.羊膜穿刺之 AFP 濃度：___________ □沒有檢查 □正常但不知道數據
□異常且不知道數據 □不確定 □不知道

27.胎兒染色體檢查結果（羊膜或絨毛採樣取得之檢體，異常才需填寫）：

____

28.母血 AFP 濃度：___________ □ 沒有檢查 □ 正常但不知道數據
□ 異常且不知道數據 □不確定 □不知道

29.產科併發症：□ 無 □有(請填至下方備註) □不確定 □不知道

30.備註（前 1~29 題若需補充說明，請簡述於此）：

____

____

____

台灣癲癇妊娠登錄系統（D 表）

（生產時完填寫生產資料，產後七天內完成新生兒資料）

※ 填表前請先確定您有填寫過收案同意書

※ 本問卷只做個案管理及追蹤，並保證不會將資料外流，請放心填寫

身份證字號：______________

為進一步了解您的健康情形，以便在懷孕的過程中提供更佳的臨床處置，請務必確實回答以下問題：

01.D 表登錄日期：西元_____年_____月_____日

02.個案登錄日期：（免填）

03.第三孕期內抽煙：□無 □1-10 隻/天 □11-20 隻/天 □ >20 隻/天 □不確定 □不知道

04.第三孕期內飲酒：□無 □<1 杯/天 □<3 杯/天 □3-6 杯/天 □>6 杯/天 □不確定□不知道

05.詳述其他第三孕期內疾病及感染：（若無則免填）______________________________

06.第三孕期內是否服用抗癲癇藥物：□否 □是 □不確定 □不知道

抗癲癇藥物（AED）	學名	每日服用總劑量(mg)	每日服用次數	開始服用日期 (yyyy/mm/dd)	結束服用日期 (yyyy/mm/dd)
07.AED1					
08.AED2					
09.AED3					
10.AED4					
11.AED5					

11 bis 第一孕期內是否有更換抗癲癇藥物種類或劑量？□無 □是 □不確定 □不知道

*****若曾變更、增加或減少抗癲癇藥物的種類或劑量，皆須記錄該種類或劑量的開始及結束服用日期*****

12.第三孕期使用的其它藥物：（若無則免填）______________________________

13.第一孕期時全身性大發作次數：（發作時為全身抽蓄、牙關緊閉等情形）

□無 □<1 次/月 □每月都有 □每週都有 □>每週 □每天都有

□其他 □不確定 □不知道

14.第一孕期時一般性發作次數：（發作時為失神性或局部抽蓄等情形）

□無 □<1 次/月 □每月都有 □每週都有 □>每週 □每天都有

□其他 □不確定 □不知道

15.第一孕期時是否曾發生癲癇重積的情形：□無 □無抽搐 □有抽搐 □不確定 □不知道
（全身性大發作時間超過 5 分鐘，且反覆發作，反覆發作的過程中皆無恢復意識）

16.生產時的併發症：□無 □有(請填至下方備註) □不確定 □不知道

17.嬰兒娩出日期：西元_____年____月____日 嬰兒娩出週數：____週

18.嬰兒性別：□男 □女 □不確定 □不知道

19.嬰兒出生地: □醫院 □家裡 □其他（請填至備註欄） □不確定 □不知道

20.嬰兒出生方式：□NSD □引產（未使用任何器械） □CS □vacuum
□forceps □其他 □不確定 □不知道

21.單胞胎或多胞胎：□單胞胎
□雙胞胎中第一個出生的孩子 □雙胞胎中第二個出生的孩子
□ 三胞胎中第一個出生的孩子 □三胞胎中第二個出生的孩子
□ 三胞胎中第三個出生的孩子 □不確定 □不知道

22.生產中產婦發生 seizure（含 status epilepticus）：□無 □抽搐發作 □其他
□有抽搐症狀 □無抽搐症狀
□不確定 □不知道

23.嬰兒出生 1 分鐘時之 Apgar score：_______ □不確定 □不知道

24.嬰兒出生 5 分鐘時之 Apgar score：_______ □不確定 □不知道

25.嬰兒出生體重：______g □不確定 □不知道

26.嬰兒出生身長：______cm □不確定 □不知道

27.嬰兒出生頭圍(occipital-frontal head circumference)：_______cm
□不確定 □不知道

28.嬰兒於周產期時死亡：□否（請跳答 31 題） □是 □不確定 □不知道

29.嬰兒於周產期時死亡之日期：西元_________年_____月_____日

30.嬰兒於周產期時死亡之原因：____

31.嬰兒是否有先天畸形：□無（請跳答 33 題） □畸形 □染色體異常
□染色體異常且畸形 □不確定 □不知道

32.描述嬰兒之先天畸形：

____

33.備註：（前 1~32 題若需補充說明，請簡述於此）：

____

____

台灣癲癇妊娠登錄系統（E 表）

（寶寶週歲時填寫）

填表前請先確定您有填寫過收案同意書

※ 本問卷只做個案管理及追蹤，並保證不會將資料外流，請放心填寫。

身份證字號：__Q223038044__

為進一步了解您的健康情形，以便在懷孕的過程中提供更佳的臨床處置，請務必確實回答以下問題：

實際小兒科就診醫院：____________________________

實際小兒科主治醫師：____________________________

01.E 表登錄日期：西元__________年______月______日

02.個案登錄日期：（免填）

03.追蹤方法：□電話　□回診　□其他：____________________________

單胞胎或多胞胎：□單胞胎
□雙胞胎中第一個出生的孩子　□雙胞胎中第二個出生的孩子
□三胞胎中第一個出生的孩子　□三胞胎中第二個出生的孩子
□三胞胎中第三個出生的孩子　□不確定　□不知道

04.新生兒死亡（未滿週歲）：□否（請跳答 6 題）　□是

05.描述新生兒死亡原因：

06.出生後發生先天性異常：□無（請跳答 9 題）　□畸形　□染色體異常
□染色體異常且畸形　□不確定　□不知道

07.描述新生兒之先天性異常：

08.幾個月時發現有先天性異常：_______個月

09.新生兒滿週歲前曾住院：□否（請跳答 11 題）　□是　□不確定　□不知道

10.描述新生兒住院的原因：

11.備註：（前 1~10 題若需補充說明，請簡述於此）
