# Supplementary material for: Antiepileptic drug use among women from the Taiwanese Registry of Epilepsy and Pregnancy: Obstetric complications and fetal malformation outcomes
Source: PLoS One. 2017 Dec 18;12(12):e0189497. doi: 10.1371/journal.pone.0189497 (PMC5734752; doi:10.1371/journal.pone.0189497)
Supplement: S1 Table — Notes. a MCM = major congenital malformation, b PDA = Patent Ductus Arteriosus, c DORV = Double outlet right ventricle. d. Live Birth: Y = live birth, N = still birth, a = aborted. e. Ultrasound Screening Performed: Y = Yes, N = No, n/a = not available. f. Positive Findings: 5/9 = 55.6% accuracy. g. Means reported for live births only. *, **, ***, †, ‡, indicate concurrent maternal complications (S2 Table). (PDF) [file pone.0189497.s003.pdf]

S1 Table. Fetal malformation listed by cases (N=16).

Case #	*Fetal Malformations Reported*	Live Birth[d]	Level 2 Ultrasound Performed[e]	Level 2 Ultrasound Finding	Positive Level 2 Ultrasound MCM[a] Findings	Gestational Age (weeks)	Birth Weight (g)
1	Atrial Septal Defect	Y (1)	Y (1)	Normal (1)	N (1)	40	3020
2	Atrial Septal Defect and Pulmonary Vein Stenosis	Y (2)	Y (2)	Single Umbilical Artery	Y (1)	38	2500
3	Bilateral Renal Agenesis	Y (3)	Y (3)	Bilateral Renal Agenesis	Y (2)	27	n/a
*4	Cardiac Malformations	Y (4)	N (1)	n/a	n/a	38	2554
5	Choledcochal Cyst	Y (5)	Y (4)	Normal (2)	N (2)	37	2880
**6	Hypospadias	Y (6)	N (2)	n/a	n/a	34	2460
7	Cleft Lip & Palate	Y (7)	Y (5)	Cleft Lip & Palate	Y (3)	38	2910
***8	Pyelactasis	Y (8)	N (3)	n/a	n/a	35	1995
†9	Renal & Cardiac Malformations, vascular ring	Y (9)	Y (6)	Normal (3)	N (3)	35	2019
10	Valve Insufficiency	Y (10)	Y (7)	Normal (4)	N (4)	36	2580
11	Ventricular Septal Defect	Y (11)	Y (8)	VSD	Y (4)	38	2820
‡12	PDA[b]	Y (12)	N (4)	n/a	n/a	38	2280
13	Anencephalus	a (1)	na (1)	n/a	n/a	15	n/a
14	Cystic Hygroma	a (2)	na (2)	n/a	n/a	14	n/a
15	Limb Deformity	a (3)	na (3)	n/a	n/a	15	n/a
16	Trisomy 21, DORV[c]	a (4)	Y (9)	DORV	Y (5)	20	n/a
	Cardiac-related malformation (7), 43.8%	**Y (12)**	**Y (9)**	***Normal (4)***	**Y (5)**[f]	**36.2 ± 0.01**[g]	**2547.1 ± 1.20**[g]
