# Supplementary material for: Antiepileptic drug use among women from the Taiwanese Registry of Epilepsy and Pregnancy: Obstetric complications and fetal malformation outcomes
Source: PLoS One. 2017 Dec 18;12(12):e0189497. doi: 10.1371/journal.pone.0189497 (PMC5734752; doi:10.1371/journal.pone.0189497)
Supplement: S2 Table — Notes. a. CS = Cesarean section, b. PPROM = Preterm premature rupture of the membranes, c. n/a = not applicable. d.Live Birth: Y = live birth, N = still birth, a = aborted. e. CS rate 11/14 = 78.5%. *, **, ***, †, ‡, indicate concurrent fetal malformations (Table 2). (PDF) [file pone.0189497.s004.pdf]

S2 Table. Obstetric complications listed by cases (N=14).

Case #	*Obstetric Complication Reported*	Live Birth[d]	Gestational Age (weeks)	Delivery Mode	Birth Weight (g)
1	Chronic Hypertension	Y (1)	37	CS[a] (1)	2622
2	Epilepsy after Delivery	Y (2)	39	CS (2)	3384
3*	Epilepsy before Delivery	Y (3)	38	CS (3)	2554
4	Gestational Diabetes Mellitus	Y (4)	37	Vaginal Delivery (1)	3214
5**	Pre-eclampsia	Y (5)	34	n/a[c]	2460
6	Gestational Hypertension (1)	Y (6)	37	CS (4)	3100
7	Gestational Hypertension (2)	Y (7)	37	CS (5)	3470
8	Gestational Hypertension (3)	Y (8)	37	CS (6)	2374
9	Preterm Birth (1)	Y (9)	36	Vaginal Delivery (2)	2580
10	Preterm Birth (2)	Y (10)	32	CS (7)	2250
11***	PPROM[b] (1)	Y (11)	35	CS (8)	1995
12	PPROM (2)	Y (12)	34	CS (9)	2520
13†	PPROM (3)	Y (13)	35	CS (10)	2019
14‡	Epilepsy in Delivery	Y (14)	38	CS (11)	2280
	Gestational Hypertension (3), PPROM (3)	***Y (14)***	***36.1 ± 0.01***	***CS (11)***[e]	***2630.1 ± 1.65***
